# Premonitory Urge and Tic Severity, Comorbidities, and Quality of Life in Chronic Tic Disorders

**DOI:** 10.1002/mdc3.13742

**Published:** 2023-04-17

**Authors:** Valerie Brandt, Jana Essing, Ewgeni Jakubovski, Kirsten Müller‐Vahl

**Affiliations:** ^1^ School of Psychology, Centre for Innovation in Mental health University of Southampton Southampton UK; ^2^ Clinic of Psychiatry, Social Psychiatry and Psychotherapy Hannover Medical School Hanover Germany

**Keywords:** Tourette syndrome, chronic tic disorder, premonitory urge (PU), quality of life, comorbidity

## Abstract

**Background:**

Tics are intimately associated with premonitory urges (PU) but knowledge about urges is still limited, with small sample sizes often limiting the generalizability of findings.

**Objectives:**

This study addressed the following open questions: (1) is tic severity associated with urge severity, (2) how common is relief, (3) which comorbidities are associated with urges, (4) are urges, tics, and comorbidities associated with lower quality of life, and (5) can complex and simple, motor and vocal tics be differentiated based on PU?

**Methods:**

N = 291 patients who reported a confirmed diagnosis of chronic primary tic disorder (age = 18–65, 24% female) filled out an online survey assessing demographic data, comorbid conditions, location, quality and intensity of PU, as well as quality of life. Every tic was recorded, and whether the patient experienced a PU, the frequency, intensity, and quality of that urge.

**Results:**

PU and tic severity were significantly associated, and 85% of urge‐related tics were followed by relief. A diagnosis of attention deficit/hyperactivity disorder (ADHD) or depression, female gender, and older age increased the likelihood of experiencing PU, while more obsessive compulsive (OCD) symptoms and younger age were associated with higher urge intensities. PU, complex vocal tics, ADHD, OCD, anxiety, and depression were related to lower quality of life. Motor and vocal, complex and simple tics did not differ regarding PU intensity, frequency, and quality, or relief.

**Conclusions:**

The results shed light on the relationship between PU, tics, comorbidities, age, gender, and quality of life in tic disorders.

Chronic primary tic disorders, including Tourette syndrome (TS), are characterized by multiple motor and/or vocal tics.^1^ A preceding sensory or premonitory urge (PU) is considered a hallmark of tics.^2^, ^3^, ^4^ The urge to tic has been compared to the urge to scratch—a build‐up in tension or an uncomfortable feeling that is relieved after an action. The estimate for the prevalence of PU varies, with approximately 77% of patients over 13 years and approximately 90% of patients over 18 years reporting to experience PU.^2^, ^5^, ^6^, ^7^, ^8^ Adults with TS report urges more frequently than children do, but it is unclear whether urges develop as a consequence of tics or precede tics in development; alternatively, young children may simply lack awareness or the ability to describe PU.^9^ A recent study of 291 patients confirmed that PU tend to occur in the same body part where a tic is about to occur.^10^ The results indicated that, like tics,^11^ urges most commonly occur in the face and head.^10^

Different qualities of urges (eg, pressure) can be differentiated, for instance with the premonitory urges for tics scale (PUTS).^3^, ^12^ There is evidence that the urge increases before a tic or a bout of tics, and that the urge then decreases in the majority of patients^13^, ^14^ but a minority experiences the reverse pattern.^14^ A recent study conducted in the same sample that is utilized in this study showed that 97% of patients who experienced urges also experienced relief for at least one of their tics.^10^ However, it has not been investigated how many tics are associated with a sense of relief on average, ie, do patients experience a sense of relief with every tic or only for a subset of tics?

Patients with TS who seek diagnosis or treatment typically have at least one comorbidity, the most common are attention deficit/hyperactivity disorder (ADHD), obsessive compulsive disorder (OCD), depression, and anxiety.^15^ Evidence on whether comorbidities are associated with urge severity has been mixed,^8^, ^12^, ^16^, ^17^, ^18^, ^19^, ^20^ specifically, it is unclear whether or not comorbid OCD, ADHD, depression, and anxiety are associated with the occurrence or intensity of PU. There is also some evidence that different urge qualities may be related to comorbidities in different ways, eg, OCD may be associated with just‐right feelings and ADHD may be related to feelings of tension.^16^ PU have been found to be associated with lower quality of life (QoL)^8^, ^21^ but it has not been investigated whether different urge qualities are associated with lower QoL.

While tics can be successfully treated with behavioral therapy,^22^, ^23^, ^24^, ^25^, ^26^ pharmacotherapy (most commonly anti‐dopaminergic drugs),^27^, ^28^ cannabis‐based medicine including tetrahydrocannabinol (THC),^29^, ^30^, ^31^ and—in rare and otherwise treatment resistant cases—with surgical therapy using deep brain stimulation (DBS),^32^ it is less clear how different treatments may affect PU.^33^

The current study aims to address whether urge severity and tic severity are associated, by using data from a survey that assessed each tic and associated PU individually. This detailed data allows us to investigate which urge qualities may be more common than others and how comorbidities are related to different urge qualities. Moreover, the sample is large enough to assess the impact of tics, urges, and comorbidities on QoL, while controlling for age and gender. Finally, the association between anti‐dopaminergic medication and urge occurrence (yes/no) and severity is investigated.

## Methods

N = 291 patients (age = 18–65 years, 24% female) who reported a confirmed diagnosis of chronic primary tic disorder and who determined this diagnosis (eg, Psychiatrist, neurologist) filled out an online survey. The data was mainly collected as part of a study that aimed to investigate how urges are distributed across the body.^10^ However, the dataset provides rich information on a number of unanswered questions in the field and thus serves for further analyses. For information on patient recruitment, survey procedure and demographic characteristics please see.^10^

### Are Urge and Tic Frequency and Intensity Associated?

Using the adult tic questionnaire (ATQ),^34^ patients reported for each single tic they experienced (1) how frequently they experienced the tic (1 = sometimes, 2 = multiple times a day, 3 = once per hour, 4 = all the time), (2) how intense the tic was (“how intense was the tic in the last week?” 1 [barely noticeable]‐4 [clearly noticeable by others, potentially painful]), (3) how frequently they experienced an urge (never, sometimes, always) with every tic, and (4) how intense this urge was (1 [very low intensity]‐11 [very high intensity]). Pearson's correlations were conducted to explore the association between these variables separately in motor and vocal tics, single and complex tics.

### How Common is a Momentary Sense of Relief after a Tic?

For each tic, patients reported whether they experienced a sense of relief (yes = 1, no = 0). The percentage of tics that were associated with a sense of relief was calculated.

### Which Urge Qualities are most Common?

Patients reported different PU qualities, reflecting the six PUTS urge quality categories^12^ (feeling itchy, pressure, tension, not just‐right, incomplete, energy) and an additional two items that break down the last PUTS item into two separate questions (the feeling that something is building before a tic, feeling discomfort) for different tics, ie, a certain quality for each tic. Whether a patient experienced a certain urge quality with at least one tic was coded as yes/no (1/0). Percentages of tics associated with different urge quality are reported overall and split by simple and complex as well as motor and vocal tics. *χ* ^2^‐square tests were run between the different urge quality categories on tics overall and corrected for multiple testing. Only *P* < 0.001 was considered significant.

A repeated measures ANOVA was conducted with the eight different urge qualities as dependent variables, in order to assess how many patients endorsed each urge quality at least once, and compare which urge quality was experience most and least often across patients. Contrasts were calculated between the different urge qualities. Mean urge intensity was calculated across tics within each patient per urge quality. Only nine patients reported experiencing every urge quality. Therefore, paired‐t‐tests were computed, comparing each urge quality to all others and these were corrected for multiple comparisons. Only *P* < 0.001 was considered significant.

### Are Comorbidities Associated with the Urge to Tic?

Comorbidities were assessed in two different ways. Patients filled out the Obsessive Compulsive Inventory‐Revised (OCI‐R),^35^ a self‐report scale with good reliability, convergent and divergent validity.^36^, ^37^ Current ADHD symptoms were assessed with the German ADHD self‐rating scale (ADHS‐SB),^38^ symptoms of depression were assessed with the Beck Depression Inventory (BDI‐II),^39^ and symptoms of anxiety were assessed using the Beck Anxiety Inventory (BAI).^40^, ^41^, ^42^ Patients were also asked to report any of the following comorbid diagnoses: ADHD (N = 41; impulsivity, hyperactivity, and inattention were also reported separately), OCD (N = 157), anxiety disorders (N = 61), depression (N = 73), sleeping disorders (N = 40), eating disorders (N = 14), personality disorders (N = 18) and addiction disorders (N = 5). The diagnoses were entered into a binary logistic regression (except addiction disorders and eating disorders, due to low prevalence) to predict the likelihood of experiencing urges. Total number of comorbidities was correlated with urge intensity.

A structural equation model (SEM) was used to test the association of ADHD, OCD, anxiety, and depression, with the number of tics in each patient that were associated with a particular urge quality.

### Symptom Severity and QoL

Linear regression was used to predict QoL from urge qualities, comorbidities, and different types of tics, age and gender. Frequency of tics in different body areas were averaged according to the following body areas: eyes, nose/lips, grimacing, mouth/jaw, head, body, arms and legs as well as when classified as complex motor/vocal tics, eg, copropraxia, echolalia.

### Treatment

Independent *t*‐tests were used to compare different current treatments anti‐dopaminergic medication (N = 126) to no treatment (N = 137). Behavioral therapy (N = 11), and cannabis‐based treatment (N = 16) were not analyzed due to low numbers.

## Results

### Is Urge Frequency and Intensity Associated with Tic Frequency and Intensity?

A significant repeated measures ANOVA *F*_(3,396)_ = 9.90, *P* < 0.001, *η* ^2^ = 0.07 showed that patients executed complex motor tics *F*_(1,132)_ = 20.49, *P* < 0.001, *η* ^2^ = 0.13, and complex vocal tics *F*_(1,132)_ = 13.28, *P* < 0.001, *η* ^2^ = 0.09 less frequently than simple motor tics, but not simple vocal tics *F*_(1,132)_ = 1.13, *P* < 0.289, *η* ^2^ = 0.01. In contrast, tic intensity did not differ significantly across simple and complex motor and vocal tics *F*_(3,396)_ = 2.42, *P* < 0.065, *η* ^2^ = 0.02.

On average, tic frequency was significantly associated with urge frequency (*r* = 0.27, *P* < 0.001), for both motor tics (*r* = 0.27, *P* < 0.001), and vocal tics (*r* = 0.30, *P* < 0.001). If the frequency of all urges and tics were considered and used as a sum score, the correlation was much higher (*r* = 0.84, *P* < 0.001). Considering all tics, more frequent motor tics (*r* = 0.82, *P* < 0.001), and more frequent vocal tics (*r* = 0.76, *P* < 0.001) were significantly associated with more frequent urges. Similar effect sizes were found for simple (*r* = 0.80, *P* < 0.001), and complex tics (*r* = 0.80, *P* < 0.001).

On average, urge intensity was associated with tic intensity (*r* = 0.37, *P* < 0.001), similar effect sizes were found for motor (*r* = 0.39, *P* < 0.001), and vocal tics (*r* = 0.47, *P* < 0.001), and for simple (*r* = 0.40, *P* < 0.001) and complex tics (*r* = 0.30, *P* < 0.001).

### How Common is a Sense of Relief?

Of those patients who reported PUs, 97% (N = 224/232) reported a feeling of relief for a least one of their tics. In those patients who did report a sense of relief, 85% of tics were associated with relief. Eighty‐eight percent of simple motor tics, 90% of complex motor tics, 86% of simple phonetic tics, and 81% of complex phonetic tics were associated with relief.

### Which Urge Qualities are most Common?

**Figure 1:**
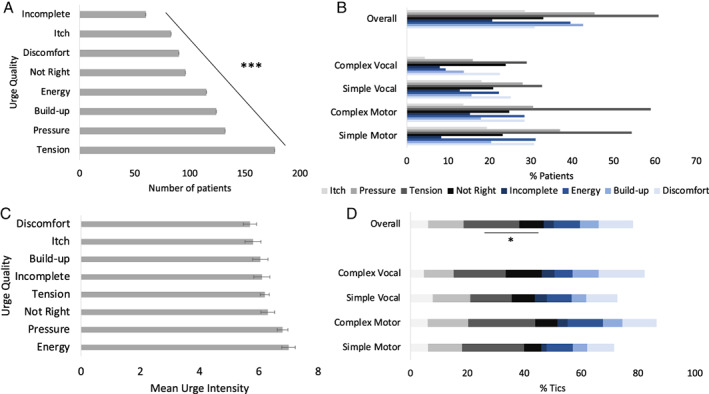
(A) Number of patients (±Standard Error) experiencing urge quality associated with at least one tic. (B) Percentage of patients reporting each urge quality for at least one tic (multiple responses possible). (C) average urge intensity associated with tics. (D) Percentage of tics associated with different types of urge quality.

Different urge qualities were experienced with decreasing frequencies of patients in the following order, in a significant linear fashion: feelings of tension, pressure and the feeling that something was building up, a feeling of energy that needs to be released, a just‐right feeling, feeling discomfort, an itch or incompleteness (Fig. 1, Table S1); *F*_(1,290)_ = 161.49, *P* < 0.001, *η* ^2^ = .28. Tension was experienced by significantly more patients than all other urge qualities (contrast between tension and pressure: *F*_(1,290)_ = 34.77, *P* < 0.001, *η* ^2^ = 0.11), and a feeling of incompleteness was experienced by significantly fewer patients than all other urge qualities (contrast between incompleteness and itch: *F*_(1,290)_ = 6.05, *P* = 0.015, *η* ^2^ = .02). The pattern was the same across simple and complex, as well as motor and vocal tics (Fig. 1B).

Urge intensity did not differ across urge qualities *F*_(1, 8)_ = 0.02, *P* = 0.896, *η* ^2^ = 0.002 (Fig. 1C).

A similar pattern was reflected in urge quality associated with tics. Most tics were associated with feelings of tension, while fewest tics were associated with a feeling of incompleteness. Correcting for multiple testing, only those two occurred at significantly different rates (*t*(290) = 10.37, *P* = 0.001, *d* = 0.61). Again, the pattern was reflected in simple and complex as well as in motor and vocal tics (Fig. 1D, Table S1).

### Are Comorbidities Associated with the Urge to Tic?

Having comorbid depression or anxiety, being older, and being female were associated with being more likely to experience urges (χ^2^ = 49.52, *P* < 0.001, Nagelkerkes *r* ^2^ = 0.25; Table 1). Having more comorbidities was not associated with more intense urges (*r* = 0.09, *P* = 0.183) but with a higher likelihood to experience urges (*r* = 0.19, *P* = 0.001). Higher urge intensity was only predicted by more OCD symptoms and younger age (Table 1).

**TABLE 1:** Regression predicting urges from comorbidities

	B	S.E.	Wald	OR	*P*
Constant	−2.83	1.15	6.04	0.06	0.014
OCD	−0.63	0.34	3.38	1.88	0.066
Anxiety	0.21	0.51	0.17	0.81	0.683
Depression	2.07	0.78	7.03	0.13	**0.008**
ADHD	2.66	1.05	6.49	0.07	**0.011**
Sleep	0.37	0.83	0.20	0.69	0.653
Personality	−0.25	0.90	0.08	1.28	0.783
Age	0.55	0.19	8.37	1.73	**0.004**
Gender	2.76	1.04	7.10	15.83	**0.008**

	B	S.E.		β	P
Constant	5.66	0.43			0.000
ADHD	0.02	0.03		0.04	0.613
OCD	0.03	0.01		0.22	**0.017**
Anxiety	0.01	0.01		0.06	0.550
Depression	0.00	0.01		−0.02	0.774
Age	−0.24	0.10		−0.16	**0.017**
Gender	0.38	0.26		0.10	0.150

*Note*: The upper panel shows a binary logistic regression with diagnosis (yes/no) as predictor variables and experiencing urges (yes/no) as the dependent variable. Having a depression or ADHD diagnosis significantly increased the likelihood to experience urges. Significant results are marked in bold. The lower panel shows the results of a linear regression, predicting urge intensity from questionnaire data. Attention deficit hyperactivity disorder (ADHD) and younger age were associated with more intense urges. OCD, obsessive compulsive disorder.

**Figure 2:**
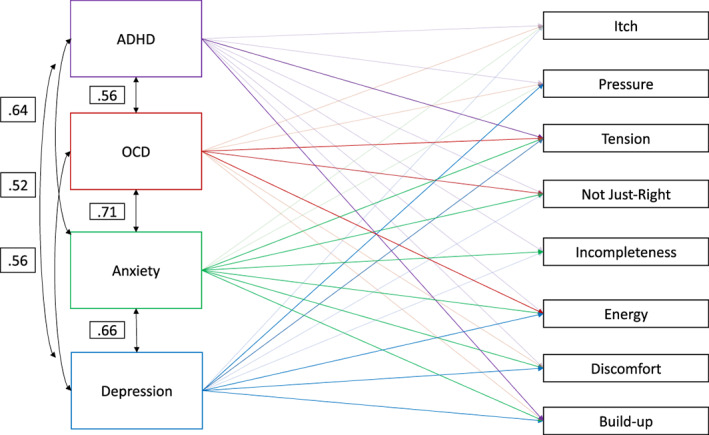
Structural equation model. Significant beta values are displayed in the model. Correlations amongst the predictors are also displayed. ADHD, attention deficit hyperactivity disorder; OCD, obsessive compulsive disorder.

The SEM showed that different comorbidities were related to experiencing more intense urges of different qualities, eg, ADHD was related to higher intensities of tension and the feeling of something building up before a tic, OCD was related to not‐just‐right feelings, tension, and energy, depression was associated with pressure, tension, energy, and the feeling that urges build up, while anxiety was associated with tension, not‐just‐right feelings, incompleteness, energy, and the feeling of something building up before a tic (Fig. 2, Table 2).

**TABLE 2:** Association between different urge qualities and comorbidities

		Estimate	S.E.	*z*	*P*
Itch					
	ADHD‐SB	0.07	0.04	1.66	0.097
	OCI‐R	0.03	0.02	1.75	0.080
	BAI	−0.03	0.02	−1.18	0.239
	BDI‐II	0.03	0.02	1.25	0.211
Pressure					
	ADHD‐SB	0.09	0.05	1.76	0.078
	OCI‐R	0.03	0.02	1.44	0.151
	BAI	0.04	0.03	1.57	0.118
	BDI‐II	0.06	0.03	2.22	**0.027**
Tension					
	ADHD‐SB	0.11	0.04	2.64	**0.008**
	OCI‐R	0.05	0.02	2.79	**0.005**
	BAI	0.05	0.02	2.03	**0.042**
	BDI‐II	0.05	0.02	2.12	**0.034**
Just right					
	ADHD‐SB	0.07	0.04	1.72	0.086
	OCI‐R	0.04	0.02	2.47	**0.014**
	BAI	0.08	0.02	4.05	**<0.001**
	BDI‐II	0.01	0.02	0.56	0.573
Incompleteness					
	ADHD‐SB	0.00	0.04	0.10	0.918
	OCI	0.02	0.02	1.12	0.263
	BAI	0.05	0.02	2.60	**0.009**
	BDI‐II	0.01	0.02	0.29	0.771
Energy					
	ADHD‐SB	0.03	0.05	0.49	0.627
	OCI‐R	0.06	0.02	2.67	**0.008**
	BAI	−0.09	0.03	−3.14	**0.002**
	BDI‐II	0.08	0.03	2.79	**0.005**
Discomfort					
	ADHD‐SB	0.08	0.05	1.76	0.079
	OCI‐R	0.00	0.02	0.10	0.924
	BAI	−0.07	0.02	−2.89	**0.004**
	BDI‐II	0.10	0.03	3.98	**<0.001**
Build‐up					
	ADHD‐SB	0.12	0.05	2.70	**0.007**
	OCI‐R	0.02	0.02	0.75	0.455
	BAI	−0.06	0.02	−2.67	**0.008**
	BDI‐II	0.07	0.02	2.77	**0.006**

*Note*: The table shows which comorbidities are associated with different urge qualities. Significant associations are highlighted in bold. ADHD‐SB, attention deficit hyperactivity disorder self‐rating scale, OCI‐R, obsessive compulsive inventory, BDI‐II, Beck Depression Inventory II, BAI, Beck Anxiety Inventory.

### Symptom Severity and QoL

Lower QoL was associated with higher urge intensity (*r* = 0.28, *P* < 0.001) and higher tic frequency (*r* = 0.13, *P* = 0.045). Linear regression showed that more complex vocal tics, and having comorbid OCD, ADHD, anxiety or depression were associated with lower QoL (*F*_(18,272)_ = 59.17, *P* < 0.001, *r* ^2^ = 0.80; Table 3).

**TABLE 3:** Variables that predict quality of life

	B	S.E.	*ß*	*t*	*P*
(Constant)	1.08	2.24		0.48	0.632
N Simple Motor Tics	0.10	0.21	0.02	0.49	0.625
N Complex Motor Tics	0.50	0.29	0.09	1.69	0.091
N Simple Vocal Tics	−0.12	0.31	−0.02	−0.39	0.699
N Complex Vocal Tics	1.85	0.55	0.14	3.34	**0.001**
ADHD‐SB	1.10	0.15	0.29	7.51	**<0.001**
OCI‐R	0.17	0.06	0.11	2.60	**0.010**
BAI	0.36	0.08	0.23	4.52	**<0.001**
BDI‐II	0.63	0.08	0.32	8.10	**<0.001**
Itch	0.47	1.38	0.01	0.34	0.734
Pressure	0.66	1.47	0.02	0.45	0.652
Tension	−0.58	1.62	−0.01	−0.36	0.720
Just right	0.20	1.58	0.00	0.12	0.901
Incomplete	−3.00	1.63	−0.06	−1.85	0.066
Energy	−0.47	1.32	−0.01	−0.35	0.725
Discomfort	0.35	1.85	0.01	0.19	0.852
Build‐up	−1.11	1.99	−0.03	−0.56	0.576
Age	−0.39	0.52	−0.02	−0.76	0.450
Gender	−0.67	1.44	−0.01	−0.47	0.641

*Note*: A linear regression showed that a higher number of complex vocal tics, and higher symptoms of attention deficit hyperactivity disorder (ADHD‐SB), obsessive compulsive disorder (OCD‐R), anxiety, and depression were associated with lower quality of life. BDI‐II, Beck Depression Inventory II, BAI, Beck Anxiety Inventory.

Tics in different body parts were not related to differences in QoL *F*_(9, 21)_ = 0.47, *P* = 0.876.

### Treatment

Patients who were taking anti‐dopaminergic medication were significantly less likely to experience an urge (N = 126) than those who were not *t*(261) = −2.92, *P* = 0.004. There were no differences in urge intensity between patients who took anti‐dopaminergic medication and those who did not receive treatment *t*(202) = −0.52, *P* = 0.605.

## Discussion

### Is Urge Frequency and Intensity Associated with Tic Frequency and Intensity?

The results show that simple tics were executed more frequently than complex tics but that tic intensity did not differ between simple and complex tics. The results confirm that urge and tic frequency and urge and tic intensity are correlated. This is an important result because previous findings were mixed regarding the association between urges and tics.^3^, ^12^, ^14^, ^17^, ^18^, ^43^, ^44^ It has been unclear whether the relationship between urges and tics was not as close as assumed or whether the instruments to measure urges and tics influenced the association found. The majority of studies have found small‐medium correlations between urges and tics,^3^, ^12^, ^17^, ^43^, ^44^ while others found no relationship.^18^ Our data from a large sample of patients clarifies that urges and tics are indeed closely linked.

The current study did not use questionnaires asking about general tic and urge frequencies across all tics. Instead, patients reported each tic and reported how frequent the tic was and how frequently the associated urge occurred. This also disentangles frequency and intensity, concepts that are convoluted when using overall questionnaire scores assessing urge or tic severity.^12^, ^45^ Regarding frequency, the association between average urge frequency and tic frequency was small‐medium but when all tics per patient were considered, the relationship was high (explained variance was 70%). Effects were of similar size for motor and vocal tics, as well as for simple and complex tics. The results confirm that there is a close association between tic and urge frequency, ie, tics that occur more frequently are also associated with frequent urges.

Regarding intensity, correlations showed a medium relationship between urge intensity and tic intensity on average. Again, this was true for motor and vocal as well as for simple and complex tics. The result suggests that more intense urges are followed by more intense tics. This leads back to the question whether an intense urge “requires” an intense tic to lead to relief or whether intense tics become associated with an intense urge to match the tic.

### How Common is a Sense of Relief?

In patients who experienced urges, over 80% of tics were associated with a sense of relief. Previous research showed that on average, urges increase before tics and decrease after a bout of tics.^13^ This pattern was found only in two thirds of patients, albeit in a small sample.^14^ Previous papers have focused on how many patients experience relief after executing a tic and have found that >80% of patients experience a sense of relief with at least one tic.^17^, ^21^, ^46^ These results show that in patients who do experience relief >80% of tics are associated with a sense of relief. Again, there were no substantial differences between simple and complex or motor and vocal tics. Therefore, it remains an open question what determines whether a tic is associated with a feeling of relief, and it poses the question of whether it is sensible to categorize tics in this manner.

### Which Urge Qualities are most Common?

Regarding urge quality, tension was the most commonly experienced PU, while incompleteness was the least common one. Again, there were no differences between vocal and motor tics, simple and complex tics. Furthermore, different urge qualities were not associated with different intensities. As far as we are aware, this is the first study to explore which urge qualities are most commonly associated with tics. It is interesting that most tics were associated with general feelings of energy, tension, and discomfort, while more specific qualities such as an itch, feelings of incompleteness and not just‐right feelings were less common. This poses a problem regarding the assessment of PU with the PUTS.^12^ The PUTS treats all qualities equally, and patients who score higher on more different qualities, receive a higher urge severity score based on the questionnaire. This would result in patients with high urge scores to have an unusual, rather than a usual presentation of urges. Future versions should disentangle urge quality and intensity.^47^

### Are Comorbidities Associated with the Urge to Tic?

When considering comorbid diagnoses, ADHD and depression were associated with a higher likelihood to experience urges, while OCD was associated with higher urge intensities in those that experienced urges. ADHD, OCD, and depression are common comorbidities in TS.^15^ Previous findings on the association between urges and comorbidities have been mixed.^12^, ^17^, ^18^, ^19^ No relationship was found between PU severity and severity of ADHD and OCD in a sample of 122 adolescents and young adults,^17^ while a study in 42 youths found significant relationships between PU and OCD, ADHD, and anxiety/depression.^12^ Significant relationships between PU and OCD symptoms and depression but not anxiety and ADHD were found in children older than 10 years,^18^ and correlations of PU intensity with OCD but not ADHD were also found in 22 adults.^16^ The results in this large sample of adults clarify that adult patients with ADHD and depression are more likely to experience PU, and that patients who do experience PU, experience higher intensities with more OCD symptoms, when age and gender effects are controlled for. Interestingly, having more comorbidities was not associated with more intense urges but it was associated with a slightly higher risk to experience urges in the first place.

Regarding urge quality, symptoms of ADHD were significantly associated with more intense feelings of tension. It might be assumed that inattention could lead to a reduced ability to perceived urges, therefore, the result is surprising. It is possible that the result reflects the difficulty to disentangle symptoms and experience of disorders that we currently regard as distinct, such as TS and ADHD. However, if ADHD patients experience an increased inner restlessness or tension overall, it may not be a distinct experience from the phenomena associated with tics.

OCD was related to more intense feelings of tension, not just‐right feeling, and energy. The results are in line with previous literature, showing that comorbid OCD was associated with not just‐right feelings and feelings of incompleteness, and comorbid ADHD was associated with feelings of tension.^16^ In addition, our results showed that anxiety was related to more intense feelings of tension, not‐just‐right feelings, incompleteness, and energy. Depression was related to more intense feelings of pressure, tension, energy and a build‐up of urges.

Further, older age and female gender were associated with a higher likelihood of experiencing urges in many^2^, ^7^, ^8^ but not all studies.^17^ This study shows that the likelihood of urges increases across adulthood as well, not only across early development. In contrast, urge intensity decreased with older age. The data suggest that if tics continue in a patient, the likelihood to experience urges increases during adulthood. This would support the view that tics drive the generation of the urge to tic over time, not the other way around. Regarding gender, there could be differences in attention to inner states^48^ or the ability to identify inner states between males and females. However, very little is known about gender differences and symptom development into older adult age in patients with TS.

### How are Urges Related to QoL?

Lower QoL was related to higher urge intensity and tic frequency. Higher symptom load for ADHD, OCD, depression, and anxiety, and more complex vocal tics were all significantly associated with lower QoL. This is in line with the literature, showing that tic severity, urges,^8^, ^21^ depression,^49^ ADHD, and complex tics^50^ are associated with lower QoL. Specific urge qualities were not associated with lower QoL once comorbidities were accounted for.

### Treatment

Anti‐dopaminergic medication was associated with a lower likelihood to experience urges but not lower urge intensity. Although no causal conclusions can be drawn due to the cross‐sectional nature of the study, it seems unlikely that only patients with a lower likelihood to experience urges would choose medication as treatment. Anti‐dopaminergic medication has a variety of effects, such as feeling sleepy or drowsy, that could affect the ability to perceive urges by decreasing interoceptive awareness.^48^ It is also possible that anti‐dopaminergic medication decreases the urge to tic but possible mechanisms need to be further investigated.

### Limitations

Patients who participated in this study reported that they were pre‐diagnosed, however, they were not seen by a clinician for this study to confirm their diagnosis. This was also true for the comorbid diagnoses that were reported by participants. However, patients were recruited via TS outpatient groups and TS advocacy groups, and patients who reported that their diagnosis was not confirmed by a clinician were excluded from the analyses. Patients received €25 for their participation, and this poses a small risk of multiple participation but we would consider that risk small, due to the time it took to fill out the questionnaire.

### Conclusions

Results from this large dataset clarify that urges and tics are closely associated, and that most tics are followed by a feeling of relief. While the likelihood to experience urges appears to increase for females, as well as across the adult lifespan, urge intensity decreases with age. QoL was impacted by PU, comorbid ADHD, OCD, anxiety, and depression, as well as the number of complex tics but not by motor tics or simple tics, confirming that as a rule, comorbid disorders and PU are more detrimental to QoL than tics are. Motor and vocal, complex and simple tics did not differ with regard to urge intensity, urge frequency, relief, and urge quality, posing the question whether it is sensible to differentiate between these specific categories regarding tic disorder diagnosis.

## Author Roles

(1) Research project: A. Conception, B. Organization, C. Execution; (2) Statistical Analysis: A. Design, B. Execution, C. Review and Critique; (3) Manuscript: A. Writing of the first draft, B. Review and Critique.

V.B.: 1A, 2A, 2B, 3A

J.E.: 1A, 1B, 1C, 2C, 3B

E.J.: 1A, 1B, 2C, 3B

K.R.M.‐V: 1A, 1B, 2C, 3B.

## Disclosures

**Ethical Compliance Statement:** The Hanover Medical School ethics committee reviewed and approved the research (7631). The research was conducted online, patients gave their informed consent to participate by ticking the consent box on the SocSci Survey platform. We confirm that we have read the Journal's position on issues involved in ethical publication and affirm that this work is consistent with those guidelines. We confirm that we have read the Journal's position on issues involved in ethical publication and affirm that this work is consistent with those guidelines.

**Funding Sources and Conflicts of Interest:** This work was partly supported by the Else Kröner‐Fresenius‐Stiftung within the KlinStrucMed programme 2017–2018 of the Hannover Biomedical Research School without being involved in the study design, the collection/analysis/in‐terpretation of the data, the writing of the report, or the publication process. No conflicts of interest to disclose.

**Financial Disclosures for Previous 12 Months:** Valerie Brandt received funding from the Academy of Medical Sciences. She has received royalties from Kohlhammer. Kirsten Müller‐Vahl has received financial or material research support from EU (FP7‐HEALTH‐2011 No. 278367, FP7‐PEOPLE‐2012‐ITN No. 316978), DFG: GZ MU 1527/3–1 and GZ MU 1527/3–2, BMBF: 01KG1421, National Institute of Mental Health (NIMH), Tourette Gesellschaft Deutschland e.V., Else‐Kröner‐Fresenius‐Stiftung, GW pharmaceuticals, Almirall Hermal GmbH, Abide Therapeutics, and Therapix Biosiences. She has received consultant's and other honoraria from Abide Therapeutics, adjupharm, Alexion, AMP Alternative Medical Products GmbH, Ingelheim International GmbH, Bionorica Ethics GmbH, CannaMedical Pharma GmbH, Canopy Grouth, Columbia Care, CTC Communications Corp., Demecan, Enua pharma, Ethypharm GmbH, Eurox Group, Global Praxis Group Limited, Lundbeck, MCI Germany, Neuraxpharm, Sanity Group, Stadapharm GmbH, Synendos Therapeutics AG, Syqe, Tilray, and Zambon. She is an advisory/scientific board member for Alexion, Branchenverband Cannabiswirtschaft e.V. (BvCW), CannaMedical Pharma GmbH, Bionorica Ethics GmbH, CannaXan GmbH, Canopy Growth, Columbia Care, Ethypharm GmbH, IMC Germany, Leafly Deutschland GmbH, Neuraxpharm, Sanity Group, Stadapharm GmbH, Synendos Therapeutics AG, Syqe Medical Ltd., Therapix Biosciences Ltd., Tilray, von Mende Marketing GmbH, Wayland Group, and Zambon. She has received speaker's fees from Aphria Deutschland GmbH, Almirall, Bedrocan, Camurus, CEREBRO SPAIN BIDCO S.L, Cogitando GmbH, Emalex, Eurox Deutschland GmbH, Ever pharma GmbH, GROW, Hessische Landesstelle für Suchtfragen e.V. (HLS), LIO Pharmaceuticals GmbH, Medizinischer Dienst Westfalen Lippe, Meinhardt Congress GmbH, PR Berater, Spectrum Therapeutics GmbH, Takeda GmbH, Tilray, and Wayland Group. She has received royalties from Deutsches Ärzteblatt, Der Neurologie und Psychiater, Elsevier, Medizinisch Wissenschaftliche Verlagsgesellschaft Berlin, and Kohlhammer. She served as a guest editor for Frontiers in Neurology on the research topic “The neurobiology and genetics of Gilles de la Tourette syndrome: new avenues through large‐scale collaborative projects”, is an associate editor for “Cannabis and Cannabinoid Research” and an Editorial Board Member of “Medical Cannabis and Cannabinoids” und “MDPI‐Reports” and a Scientific board member for “Zeitschrift für Allgemeinmedizin”.

## Supporting information


**Table S1.** Quality of urges: *N* of patients. The first row shows urge quality per patient for at least one tic. Second row: number of tics associated with different urge qualities. SD = standard deviation. Lower part: total number and percentages of tics associated with different urge qualities.
