# Supplementary material for: Premonitory Urge and Tic Severity, Comorbidities, and Quality of Life in Chronic Tic Disorders
Source: Mov Disord Clin Pract. 2023 Apr 17;10(6):922–32. doi: 10.1002/mdc3.13742 (PMC10272904; doi:10.1002/mdc3.13742)
Supplement: Supplementary file 1 — Table S1. Quality of urges: N of patients. The first row shows urge quality per patient for at least one tic. Second row: number of tics associated with different urge qualities. SD = standard deviation. Lower part: total number and percentages of tics associated with different urge qualities. [file MDC3-10-922-s001.docx]

| Supplementary Table 1. Quality of urges: N of patients | | | | | | | | | | |
| --- | --- | --- | --- | --- | --- | --- | --- | --- | --- | --- |
|  | Itch | Pressure | Tension | | Not Right | In-complete | Energy | Rising | | Dis-comfort |
| N (%) patients | 83 (29) | 132 (45) | 177 (61) | | 96 (33) | 60 (21) | 115 (40) | 90 (31) | | 124 (43) |
| N tics Mean (SD) | 3.3 (5.9) | 4.1 (5.1) | 4.9 (4.5) | | 3.3 (2.8) | 2.1 (1.8) | 3.6 (3.4) | 2.7 (3.0) | | 3.7 (4.3) |
| Sum tics | 272 | 539 | 870 | | 313 | 124 | 409 | 246 | | 460 |
| Intensity | 5.8 (2.4) | 6.8 (2.0) | 6.2 (2.0) | | 6.3 (2.2) | 6.1 (2.1) | 7.0 (2.4) | 6.1 (2.5) | | 5.7 (2.4) |
| QoL *r*  *Controlled for age & gender* | .18** | .36*** | .42*** | | .46*** | .24*** | .12* | .10 | | .14* |
| Quality of urges: N of Tics | | | | | | | | | | |
| Simple motor N (%) | 131 (6) | 255 (12) | 463 (22) | 128 (6) | | 39 (2) | 196 (9) | 107 (5) | 201 (9) | |
| Complex motor N (%) | 57 (6) | 134 (14) | 221 (24) | 73 (8) | | 34 (4) | 116 (12) | 65 (7) | 112 (12) | |
| Simple vocal N (%) | 69 (8) | 117 (13) | 128 (15) | 72 (8) | | 37 (4) | 77 (9) | 45 (5) | 96 (11) | |
| Complex vocal N (%) | 15 (5) | 33 (10) | 58 (18) | 40 (13) | | 14 (4) | 20 (6) | 29 (9) | 51 (16) | |
| QoL *r*  *Controlled for age & gender* | .18** | .27*** | .22*** | .36*** | | .12* | .10 | .15* | .14* | |

The first row shows urge quality per patient for at least one tic. Second row: number of tics associated with different urge qualities. SD=standard deviation. Lower part: total number and percentages of tics associated with different urge qualities.
